# What Is the Role of the Placebo Effect for Pain Relief in Neurorehabilitation? Clinical Implications From the Italian Consensus Conference on Pain in Neurorehabilitation

**DOI:** 10.3389/fneur.2018.00310

**Published:** 2018-05-18

**Authors:** Gianluca Castelnuovo, Emanuele Maria Giusti, Gian Mauro Manzoni, Donatella Saviola, Samantha Gabrielli, Marco Lacerenza, Giada Pietrabissa, Roberto Cattivelli, Chiara Anna Maria Spatola, Alessandro Rossi, Giorgia Varallo, Margherita Novelli, Valentina Villa, Francesca Luzzati, Andrea Cottini, Carlo Lai, Eleonora Volpato, Cesare Cavalera, Francesco Pagnini, Valentina Tesio, Lorys Castelli, Mario Tavola, Riccardo Torta, Marco Arreghini, Loredana Zanini, Amelia Brunani, Ionathan Seitanidis, Giuseppe Ventura, Paolo Capodaglio, Guido Edoardo D’Aniello, Federica Scarpina, Andrea Brioschi, Matteo Bigoni, Lorenzo Priano, Alessandro Mauro, Giuseppe Riva, Daniele Di Lernia, Claudia Repetto, Camillo Regalia, Enrico Molinari, Paolo Notaro, Stefano Paolucci, Giorgio Sandrini, Susan Simpson, Brenda Kay Wiederhold, Santino Gaudio, Jeffrey B. Jackson, Stefano Tamburin, Fabrizio Benedetti

**Affiliations:** ^1^Istituto Auxologico Italiano IRCCS, Psychology Research Laboratory, San Giuseppe Hospital, Verbania, Italy; ^2^Department of Psychology, Catholic University of Milan, Milan, Italy; ^3^Faculty of Psychology, eCampus University, Novedrate, Italy; ^4^Cardinal Ferrari Rehabilitation Center, Santo Stefano Rehabilitation Istitute, Fontanellato, Italy; ^5^Pain Medicine Center, San Pio X Clinic, Humanitas, Milan, Italy; ^6^IRCCS Galeazzi Orthopedic Institute, Milan, Italy; ^7^Department of Dynamic and Clinical Psychology, Sapienza University of Rome, Rome, Italy; ^8^HD Respiratory Rehabilitation Unit, IRCCS Fondazione Don Carlo Gnocchi, Milan, Italy; ^9^Department of Psychology, Harvard University, Cambridge, MA, United States; ^10^Department of Psychology, University of Turin, Turin, Italy; ^11^Anesthesia and Intensive Care, ASST Lecco, Lecco, Italy; ^12^Department of Neuroscience "Rita Levi Montalcini", University of Turin, Turin, Italy; ^13^Istituto Auxologico Italiano IRCCS, Rehabilitation Unit, San Giuseppe Hospital, Verbania, Italy; ^14^Istituto Auxologico Italiano IRCCS, Department of Neurology and Neurorehabilitation, San Giuseppe Hospital, Verbania, Italy; ^15^Pain Medicine, Anesthesiology Department, A.O. Ospedale Niguarda ca Granda, Milan, Italy; ^16^Fondazione Santa Lucia IRCCS, Rome, Italy; ^17^C. Mondino National Neurological Institute, Pavia, Italy; ^18^Department of Brain and Behavioral Sciences, University of Pavia, Pavia, Italy; ^19^University of South Australia, Adelaide, SA, Australia; ^20^Regional Eating Disorders Unit, NHS Lothian, Livingston, United Kingdom; ^21^Virtual Reality Medical Institute, Brussels, Belgium; ^22^Department of Neuroscience, Functional Pharmacology, Uppsala University, Uppsala, Sweden; ^23^Virginia Tech, Falls Church, VA, United States; ^24^Department of Neurosciences, Biomedicine and Movement Sciences, University of Verona, Verona, Italy

**Keywords:** neurorehabilitation, placebo, pain, clinical psychology, health psychology, placebo effect, consensus conference

## Abstract

**Background:**

It is increasingly acknowledged that the outcomes of medical treatments are influenced by the context of the clinical encounter through the mechanisms of the placebo effect. The phenomenon of placebo analgesia might be exploited to maximize the efficacy of neurorehabilitation treatments. Since its intensity varies across neurological disorders, the Italian Consensus Conference on Pain in Neurorehabilitation (ICCP) summarized the studies on this field to provide guidance on its use.

**Methods:**

A review of the existing reviews and meta-analyses was performed to assess the magnitude of the placebo effect in disorders that may undergo neurorehabilitation treatment. The search was performed on Pubmed using placebo, pain, and the names of neurological disorders as keywords. Methodological quality was assessed using a pre-existing checklist. Data about the magnitude of the placebo effect were extracted from the included reviews and were commented in a narrative form.

**Results:**

11 articles were included in this review. Placebo treatments showed weak effects in central neuropathic pain (pain reduction from 0.44 to 0.66 on a 0–10 scale) and moderate effects in postherpetic neuralgia (1.16), in diabetic peripheral neuropathy (1.45), and in pain associated to HIV (1.82). Moderate effects were also found on pain due to fibromyalgia and migraine; only weak short-term effects were found in complex regional pain syndrome. Confounding variables might have influenced these results.

**Clinical implications:**

These estimates should be interpreted with caution, but underscore that the placebo effect can be exploited in neurorehabilitation programs. It is not necessary to conceal its use from the patient. Knowledge of placebo mechanisms can be used to shape the doctor–patient relationship, to reduce the use of analgesic drugs and to train the patient to become an active agent of the therapy.

## Introduction

The placebo effect can be defined as the improvement in the patient’s symptoms after the administration of an inert substance in a context inducing positive expectations about its effects (1, 2). This phenomenon is raising a growing interest in the field of pain management in patients with neurological disorders. Neurorehabilitation treatments could be delayed or hampered by pain symptoms, whose management could be particularly difficult since the available treatments may provide only a moderate relief at the cost of various undesirable side effects (3–5). In this context, knowledge of the mechanisms of the placebo effect could be important. Rather than representing an alternative treatment modality, this phenomenon can be exploited to enhance the effectiveness of the care (6).

In the last decades, research has shifted its focus from the inert substance to the psychosocial context surrounding its administration. The placebo response can be considered as a form of *contextual healing*, since the beneficial outcome is due to the context of the clinical encounter, rather than to a specific efficacy of the actual treatment (7–9). This complex phenomenon can be described as the emerging effect of the doctor–patient relationship and of the psychosocial context in which it takes place (10). The patient’s memory of previous treatments, personal characteristics, and expectations modulate and are modulated by the interaction with the doctor, whose characteristics and expectations, in turn, influence the context of the encounter. Therefore, the therapeutic ritual itself is the trigger of the placebo effect (11).

The placebo effect is grounded in physiological mechanisms. Different processes can be involved, depending both on the physical or psychological state of the patient and on the context. Various theoretical frameworks have been proposed to understand them, each focusing on a different set of variables, such as conditioning processes, patient expectations, individual attributions, and contextual factors (2, 12). Each of these processes was found to involve different neurobiological mechanisms, including opioid, endocannabinoid, or dopamine ones (13–20). The presence of various mechanisms seems to reflect the complexity of the phenomenon, as well as the variety of neurobiological, psychological, and psychosocial processes involved.

The placebo effect varies across individuals and disorders. Studies are increasingly shedding some light on the individual differences, focusing on the role of genetics (21–23), on differences in the activation of the reward system (16), on differences in expectancy mechanisms and in the emotional appraisal of situations (24), or on the role of psychological variables. Among them, preliminary data corroborate the role of dispositional optimism and state anxiety (25–27), various personality traits (28, 29), hypnotizability and suggestibility (30, 31), reappraisal ability (32), beliefs (33), learning mechanisms (34), and traits linked to dopaminergic mechanisms such as novelty seeking (35).

On the other hand, differences across disorders have received less attention, especially in the field of neurorehabilitation. To exploit the analgesic potential of placebo treatments in this field, knowledge about its differential effects is required. On behalf of The Italian Consensus Conference on Pain in Neurorehabilitation (ICCPN), a multidisciplinary board aimed at developing the national guidelines on the assessment and treatment of pain in neurorehabilitation, our working group was established to summarize the available studies on this topic.

## Methods

A review of the existing reviews and meta-analyses examining the role of the placebo effect in disorders that may undergo neurorehabilitation treatment was performed. This research design was chosen since (a) it allowed to summarize a high amount of studies on such a broad topic and (b) literature reviews focusing on each disorder were already present. Both systematic and non-systematic reviews were considered for inclusion since it was hypothesized that the quality of the existing literature about each disorder would be heterogeneous. Studies were, therefore, included if they reported reviews, with or without meta-analysis, presenting data about the effects of a placebo treatment on pain intensity in disorders that may undergo neurorehabilitation treatment. Only articles written in English language were considered. Studies were excluded if they did not report summary data about the effects of placebo treatments.

An initial search was performed on July 2014, imposing no restraints on the articles’ publication date. Subsequently, a research update took place on March 2017, restraining the search to articles published from 2014 to 2017. Both the searches were performed on PubMed using the following keywords: “placebo” (research restricted to the title) “nervous system disease” (as a MeSH word), the names of the primary neurological disorders and “pain.” The inclusion and exclusion criteria were used by one of the authors to judge the eligibility of the studies based on the articles’ titles, abstracts and, finally, full texts. The bibliographies of the selected articles were analyzed to identify other potentially relevant reviews. The methodological quality of included studies was then assessed using the Critical Appraisal Checklist for Systematic Reviews (Table 1) (36). When assessing the methodological quality of non-systematic reviews, items from 5 to 8 of this checklist were not considered.

**Table 1:** Critical Appraisal Checklist for Systematic Reviews.

1	Is the review question clearly and explicitly stated?
2	Were the inclusion criteria appropriate for the review question?
3	Was the search strategy appropriate?
4	Were the sources and resources used to search for studies adequate?
5	Were the criteria for appraising studies appropriate?
6	Was critical appraisal conducted by two or more reviewers independently?
7	Were the methods used to combine studies appropriate?
8	Was the likelihood of publication bias assessed?
9	Were recommendations for policy and/or practice supported by the reported data?
10	Were the specific directives for new research appropriate?

The following data were extracted from the included reviews: study design of the review, disorder addressed by the review, participants’ details, study design of the included studies, number of electronic databases accessed during the search, date range of the search, number of studies included, number of subjects included in placebo arms, total number of subjects, instruments used by the studies to assess pain intensity, and quantitative results. Since the aim of the present review was not to assess if placebo treatments are evidence-based interventions, the quality of evidence was not graded and no recommendations were made. Instead, the results of the reviews were synthesized in a narrative form. Results from excluded reviews or from primary studies that were found during the search that were considered relevant to give insight to areas not explored by the included reviews were also commented.

## Results

**Figure 1:**
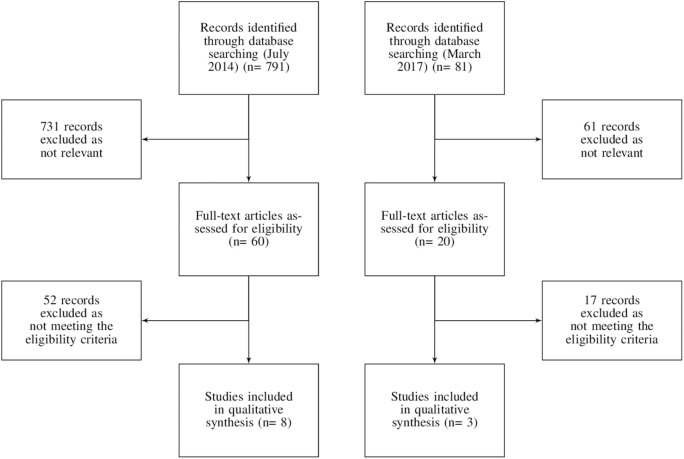
Flowchart of the records search and selection.

Overall, the searches yielded 872 records. From this sample, 11 reviews were included in the present review. The flowchart of the study search and selection is reported in Figure 1.

Among the included reviews, 10 out of 11 were systematic and 9 included a meta-analysis. Five of these reviews focused on peripheral and/or central neuropathic pain disorders, three on migraine and the remaining on chronic regional pain syndrome, fibromyalgia, or mixed chronic pain conditions. The characteristics of the studies are reported in Table 2.

**Table 2:** Description of the included reviews.

Reference	Type of review	Disorder	Participants details	Study design of the included studies	Number of databases searched	Date range of the database search	Number of trials included	Number of subjects in placebo arms	Total number of subjects
Mbizvo et al. (37)	Meta-analysis	Complex regional pain Syndrome	Patients with complex regional pain syndrome I and II	RCT and controlled studies	5 (+ other sources)	1966–2013	20 (18 included in the meta-analysis)	340	Not reported

Hauser et al. (38)	Meta-analysis	Fibromyalgia	Patients with fibromyalgia, both males and females	Double-blind RCT	3	up to 2012	18	3,546	6,589

Loder et al. (39)	Meta-analysis	Migraine	Acute migraine sufferers	RCT and controlled trials	1	1991–2002	31	Not reported	Not reported

Macedo et al. (40)	Meta-analysis	Migraine	Migraine sufferers	Double-blind RCT	1	1998–2004	32 (22 included in the meta-analysis)	1,416	4,519

Meissner et al. (41)	Meta-analysis	Migraine	Migraine sufferers	RCT	4	up to 2012	102 (79 included in the meta-analysis)	Not reported	9,287

Cragg et al. (42)	Meta-analysis	Neuropathic pain (central)	Patients with spinal cord injury, stroke or multiple sclerosis	Placebo-controlled trials	1	up to 2015	39	1,153	Not reported

Arakawa et al. (43)	Meta-analysis	Neuropathic pain	Patients with peripheral or central neuropathic pain	Placebo-controlled trials	3	1995–2014	71	Not reported	6,126

Cepeda et al. (44)	Meta-analysis	Neuropathic pain	Diabetic neuropathy, postherpetic neuralgia, central neuropathic pain, HIV-associated neuropathic pain	RCT	1 (+ other sources)	1995–2009	141	Not reported	6,239

Quessy and Rowbotham (45)	Topical review	Neuropathic pain	Patients with painful diabetic neuropathy or postherpetic neuralgia	RCT	Not reported	Not reported	35	3,355	Not reported

Tuttle et al. (46)	Systematic review	Neuropathic pain	Patients with various types of neuropathic pain	Double-blind RCT	3	1980–2013	84	Not reported	Not reported

Madsen et al. (47)	Meta-analysis	Chronic pain	Patients with headache, migraine, osteoarthritis, low back pain, postoperative pain, colonoscopy, fibromyalgia or scar pain	3-armed RCT	5	up to 2008	13	943	3,025

*RCT, randomized controlled trials*.

The methodological quality of the included reviews was variable (Table 3). Among the systematic reviews, three studies did not meet at least six items of the critical appraisal checklist (39, 40, 44), but none of them showed substantial biases that may hinder the interpretation of their results.

**Table 3:** Quality assessment of the included reviews.

Reference	Item 1	Item2	Item 3	Item 4	Item 5	Item 6	Item 7	Item 8	Item 9	Item 10
Mbizvo et al. (37)	✓	✓	✓	✓	✓	✓	✓	✓	✓	✓
Hauser et al. (38)	✓	✓	✓	✓	✗	✗	✓	✗	✓	✓
Loder et al. (39)	✓	✗	✗	✗	✓	✗	✓	✗	✓	✗
Macedo et al. (40)	✓	✓	✓	✗	✗	✗	✓	✗	✗	✗
Meissner et al. (41)	✓	✓	✓	✓	✓	✓	✓	✗	✓	✓
Cragg et al. (42)	✓	✓	✓	✗	✗	✗	✓	✓	✓	✓
Arakawa et al. (43)	✓	✓	✓	✓	✗	✗	✓	✗	✓	✓
Cepeda et al. (44)	✓	✓	✓	✗	✗	✗	✓	✗	✓	✗
Quessy and Rowbotham (45)	✗	✓	✗	✗	n\a	n\a	n\a	n\a	✓	✗
Tuttle et al. (46)	✓	✓	✓	✓	✗	✗	✓	✓	✓	✓
Madsen et al. (47)	✓	✓	✓	✓	✓	✓	✓	✗	✓	✓

## Placebo Effect in Pain Conditions in Neurorehabilitation

The main quantitative results of the included reviews generally show that the placebo effect has a low to moderate effect on pain across the various disorders (Table 4). However, differences are visible, especially when neuropathic and non-neuropathic pain disorders are contrasted.

**Table 4:** Quantitative findings of the included reviews.

Reference	Disorder	Instruments or outcome data	Results	Comments
Mbizvo et al. (37)	Complex Regional Pain Syndrome	VAS, NRS (if other scales were used, scores were converted)	Mean change in 0–100 VAS or NRS at 15–30 min posttreatment: 18,423, 95% CI [−33.15, −3.65] Mean change in 0–100 VAS or NRS at 1 week posttreatment: −6,772, 95% CI [−14.92, 1.38] Mean change in 0–100 VAS or NRS at 3–4 weeks posttreatment: 0.326, 95% CI [−2.329, 2.981] Mean change in 0–100 VAS or NRS at 6 weeks posttreatment: −3.87, 95% CI [−9.48, 1.71]	Only mean change at 15–30 min posttreatment was significant. Study design and invasiveness of the placebo treatment affected placebo response

Hauser et al. (38)	Fibromyalgia	0–10 or 0–100 VAS or NRS	Percentage of patients with a 50% reduction of pain intensity in placebo arms: 18.6%, 95% CI [17.4–19.9] vs 31.6%, 95% CI [30.5, 32.7] of patients in active drugs arms	Nocebo effect: dropout due to adverse events: 10.9%, 95% CI [9.9–11.9]. Confounders: percentage of women or Caucasians included and study duration are positively correlated with placebo effect, number of continents is negatively associated with placebo effect

Loder et al. (39)	Migraine	Percentage of pain-free patients, response rate, adverse events rate	Percentage of pain-free patients at two hours: 6.08% (±4.83) Response rate at 2 h: 28.90% (±8.55) Adverse events rate at 2 h: 23.4% (±14.05)	Migraine prophylaxis: percentage of pain-free patients: 6.02%; response rate: 25.52%; adverse events rate: 19.56%

Macedo et al. (40)	Migraine	Percentage of improved patients, attacks per month reduction, adverse events rate	Migraine prophylaxis: percentage of improved patients: 21%, 95% CI [13%, 28%] Attacks per month reduction: −0.8, 95% CI [0.4, 1.1] Adverse events rate: 30%, 95% CI [17%, 43%]	Significant confounders: study design and country

Meissner et al. (41)	Migraine	Proportion of responders (directly extracted or calculated from: number of days with migraine, number of days with headache, or 50% decrease in headache scales scores)	Proportion of placebo responders at 3–4 months: 0.26, 95% CI [0.22, 0.30] vs responders to active treatments: 0.42, 95% CI [0.38, 0.45] Difference between placebo treatments: sham surgery: 0.58, 95% CI [0.37, 0.77]; sham acupuncture: 0.38, 95% CI [0.30, 0.47]; oral placebo: 0.22, 95% CI [0.17, 0.28]	Subgroup analysis: when all confounders are considered, blinding of subjects and type of placebo treatment is positively correlated with placebo magnitude

Cragg et al. (42)	Neuropathic pain (central)	VAS, NRS	Overall mean change in pain rating (0–10): −0.64 95% CI [−0.83, −0.45]	Meta-regression: weaker placebo effect associated with higher chronic pain duration, baseline pain variability, cross-over study design. High heterogeneity

Arakawa et al. (43)	Neuropathic pain	VAS, NRS	Neuropathic pain (both central and peripheral): percentage of patients with 50% pain intensity reduction: 23%, 95% CI [20%, 25%], percentage of patients with 30% pain intensity reduction: 37%, 95% CI [34%, 41%] Peripheral neuropathic pain: percentage of patients with 50% pain intensity reduction: 23%, 95% CI [21, 26], percentage of patients with 30% pain intensity reduction: 39%, 95% CI [34%, 42%] Central neuropathic pain: percentage of patients with 50% pain intensity reduction: 14%, 95% CI [10, 19], percentage of patients with 30% pain intensity reduction: 26%, 95% CI [19%, 33%]	Among the results of the multivariable analysis, baseline pain intensity was found to be negatively correlated with placebo response in postherpetic neuralgia and in painful diabetic peripheral neuropathy

Cepeda et al. (44)	Neuropathic pain	Mean decrease in 0–10 pain intensity, responder rate (percentage of patients with 50% pain intensity reduction)	Overall: 1.2 (±1.0) mean reduction in pain intensity, RR 17% (range: 0–43%) Diabetic neuropathy: 1.45, 95% CI [1.35–1.55] mean reduction in pain intensity, RR of 20.2%, 95% CI [14.6–25.8] Postherpetic neuralgia: 1.16, 95% CI [1.03–1.29] mean reduction in pain intensity, RR of 11.5%, 95% CI [8.4–14.5] Central neuropathic pain 0.53, 95% CI [0.19–0.86] mean reduction in pain intensity, RR of 7.2%, 95% CI [2.1–12.3] HIV-associated neuropathic pain: 1.82, 95% CI [1.51–2.12], RR of 42.8%, 95% CI [34.9–50.7]	Trials evaluating NMDA blockers showed weaker placebo response, age was positively correlated with placebo response

Quessy and Rowbotham (45)	Neuropathic pain	VAS, NRS	Median change in pain intensity in PDN: 26% (range 11–35%); in PHN 15–16 (range 4–44%)	The placebo response was found to vary throughout the time course of trials and to be influenced by trial duration

Tuttle et al. (46)	Neuropathic pain	VAS, NRS	Mean change in pain intensity: 18.3%, 95% CI [15.2%, 21.4%]	Multivariable analysis: placebo magnitude is positively correlated with sample size; in studies performed in the US the placebo magnitude is positively correlated with study duration

Madsen et al. (47)	Various types of pain	SMD based on WOMAC, VAS or Likert-type rating scales	Acupuncture vs placebo SMD: −0.17, 95% CI [−0.26, −0.08]; placebo vs nontreated controls SMD: −0.42, 95% CI [−0.60, −0.23]	High heterogeneity

*VAS, Visual Analogue Scale; NRS, Numeric Rating Scale; MPQ, McGill Pain Questionnaire; WOMAC, Western Ontario and McMaster Universities Osteoarthritis Index Pain Score; CI, Confidence Interval; SMD, standardized mean difference; RR, responders rate*.

Various reviews and meta-analyses addressed the role of placebo in neuropathic pain disorders and found a noticeable heterogeneity between peripheral and central ones (43–46). In general, the placebo effect was found to be more intense in the former than in the latter. A meta-analytic study estimated the intensity of the placebo effect in various neuropathic pain disorders, and explored both the average pain reduction and the percentage of patients who positively responded to the placebo treatment (44). On a 0–10 scale, the average decrease in pain severity was 1.82 in pain associated with HIV (percentage of positive responders: 48.2%), 1.45 in painful diabetic peripheral neuropathy (percentage of positive responders: 20%), 1.16 in postherpetic neuralgia (percentage of positive responders: 11.5%), and between 0.44 and 0.64 in central neuropathic pain (percentage of positive responders: 7.2%) (42, 44). Other studies confirmed that, among the disorders associated with peripheral neuropathic pain, the placebo effect was higher in painful diabetic peripheral neuropathy than in postherpetic neuralgia (43). In contrast, the placebo effect in complex regional pain syndrome, a disorder with some neuropathic characteristics (48), seems to be nearly absent, with only weak short-term effects (37). In neuropathic pain disorders, the intensity of the placebo effect is modulated by the duration of treatment, with longer treatments associated with increased effects, and by the duration and intensity of initial pain, with longer duration of and higher intensity associated with a reduced placebo response (42, 43, 45).

The intensity of the placebo effect is generally higher in non-neuropathic pain disorders. A meta-analysis by Madsen et al. (47) compared the effects of acupuncture, placebo acupuncture, and a no-treatment condition on pain from various disorders, including headache (tension type, migraine), nociceptive pain (osteoarthritis, low back pain), iatrogenic pain (postoperative, procedural pain during colonoscopy, abdominal scar pain), and fibromyalgia. In this study, acupuncture was found to have slightly stronger effects (i.e., 0.4 points on a 0–10 scale) than placebo acupuncture, whereas a moderate difference (i.e., 1.0 points) was found between placebo acupuncture and no acupuncture conditions.

Hauser et al. (38) studied both the placebo and the nocebo effect in the management of fibromyalgia, and estimated that the percentage of patients experiencing a 50% pain reduction after a placebo treatment was 18.6% and that the dropout rate due to adverse events was 10.9%. In contrast, groups receiving a true drug showed a higher rate of responders (31.6%) and a higher dropout rate due to adverse events (20.4%). This study did not compare the improvement in the placebo group with that in the untreated groups.

Despite the variability of their effects, placebo treatments were found to be associated with both short- and long-term improvements in migraine sufferers (39, 41). Placebo groups showed an improvement of pain symptoms in 26% of cases, and 21% of patients taking placebo for migraine prophylaxis improved. For both outcomes, the efficacy of the placebo treatment was estimated to be half of that of active drugs. The placebo treatment type influenced its efficacy, with sham acupuncture and sham surgery being more effective than oral placebos (41). These effects were accompanied by a high rate of adverse events (39–41). The presence of adverse events in case of placebo administration is in line with the nature of placebo, since their characteristics are generally similar to the characteristics of the active drugs against which placebo is compared (49).

The size of the placebo effect and its variability across disorders and type of placebo treatment is apparent also when non-neurological disorders are considered. It was estimated that placebo treatments for osteoarthritis resulted in an overall moderate effect (effect size = 0.51) and that topical and intra-articular placebos are more effective than oral ones (effect size differences of 0.20 and 0.29, respectively) (50, 51). Other estimates show that the size of the placebo effect is equivalent to 72% of that of the drug treatment in burning mouth syndrome (52) and that it leads to pain remission rates of 19.9% in chronic pancreatitis (53).

## Implications for Clinical Practice

The effectiveness of placebo treatments should not be overestimated. Most of the studies on this topic showed high heterogeneity and did not take into account confounding variables, such as spontaneous remission of symptoms or regression toward the mean, thus potentially overestimating the intensity of the placebo response. In addition, various authors underlined that (a) placebo effect is higher when subjective rather than objective outcome measures are explored (54); (b) bias may be present in patients’ responses (54); (c) each individual responds differently to placebos (55, 56), and (d) outcomes vary consistently across studies and methodological design (57). These limitations are prominent in studies on placebo treatments, and may impede to predict their effects in routine clinical practice. It is recommended to take these treatments into consideration in neurorehabilitation settings only after traditional ones have failed or are contraindicated (58–64).

Rather than simply representing an alternative type of treatment, the placebo effect is a phenomenon that can increase the effectiveness of the care, since it constitutes the process through which the doctor–patient relationship becomes therapeutic. The knowledge of relevance of the placebo effect for each specific pain disorder is recommended to exploit its potential. For example, placebo response is generally small in central neuropathic pain, where pharmacological and non-pharmacological treatments have also limited efficacy, while it appears to represent half of the effect of active treatments in the prophylaxis of primary headaches. This information is central to shape the communication with the patient, allowing to provide a trustworthy explanation of the positive effects of the therapeutic context.

It is increasingly acknowledged that concealment is not necessary for the placebo effect to take place. Research on open-label placebos treatments, i.e., non-deceptive treatments in which the participants are alerted that the therapeutic mean is inert, but are informed about the effects of the administration of placebos, corroborates this claim. Further studies are needed, but open-label placebo treatments seem to have a similar or even higher efficacy than deceptive ones and are associated with marked improvement of symptoms of a variety of conditions (65–70). These treatments are more easily accepted by patients (71) and overcome the ethical and legal implications of the deceitful prescription of placebos, which violates the principle of the informed consent and may affect the trust that shape the doctor–patient relationship (72, 73).

Various techniques can be used to improve the patient’s symptoms through placebo mechanisms. A possible strategy is to maximize the patient’s expectations regarding the treatment. This can be done by informing the patient on the nature and effects of placebo analgesia, by assessing the appropriateness of the patient’s beliefs about his disorder and its treatment and providing information in case they are excessively positive or negative. In this case, it would be important to balance the information regarding the positive and negative effects of the treatment, underlying the role of the positive ones despite its undesired effects, and by cognitively reinforcing the impact of the positive outcomes as they appear (74–76). Furthermore, it is possible to exploit conditioning mechanisms to support the pharmacological therapy. Once the person associates the characteristics of the analgesic agent, such as appearance and taste, to the reduction of pain, it could be possible to employ inert substitutes with the same characteristics to obtain similar results (6). Using similar methods, it would be possible, after an adequate initial conditioning, to progressively reduce the administration of medication by alternatively switching to a placebo with similar characteristics (74, 77). Finally, the patient can also be trained to create those conditions that maximize the placebo effect, for example by focusing on the characteristics of the analgesic agent or by increasing his own expectations through appropriate information (75).

It should be underscored that all these techniques need to take place within the context of a doctor–patient relationship. The relational aspect of the placebo effect resides in the person’s feeling of being taken care for and in the process by which he himself becomes an active agent of the therapy (78). Having an empathic attitude, reassuring the patient, helping him to self-manage his symptoms, emphasizing the role of interpersonal resources and creating therapeutic rituals during therapy represent key aspects of the relationship.

In conclusion, the neurorehabilitation team needs to address a variety of disorders, each of which responds differently to the placebo effect. It is, therefore, necessary to personalize all these features depending on the disorder and on the patient’s characteristics. Studies are beginning to clarify the genetic, biological, psychological, and contextual factors that may enable to identify subjects with high or low likelihood of experiencing a placebo response (22, 28). To exploit the placebo effect, the doctor should collect information regarding not only about the patient’s disorder, but also about his personal characteristics and his context (74). The context of the doctor–patient relationship should be shaped so that the doctor does not focus only on the treatment of pain as a symptom of the neurological disorder, but is able to take care of the person as a whole.

## The Italian Consensus Conference on Pain in Neurorehabilitation

The following Authors, who are listed in alphabetical order, contributed to the work of the Italian Consensus Conference on Pain in Neurorehabilitation: **Michela Agostini**, Neurorehabilitation Department, Foundation IRCCS San Camillo Hospital, Venice, Italy; **Enrico Alfonsi**, C. Mondino National Institute of Neurology Foundation, IRCCS, Pavia, Italy; **Anna Maria Aloisi**, Department of Medicine, Surgery and Neuroscience, University of Siena, Siena, Italy; **Elena Alvisi**, Department of Brain and Behavioural Sciences, University of Pavia, Pavia, Italy; **Irene Aprile**, Don Gnocchi Foundation, Milan, Italy; **Michela Armando**, Department of Neuroscience and Neurorehabilitation, Bambin Gesù Children’s Hospital, IRCCS, Rome, Italy; **Micol Avenali**, C. Mondino National Institute of Neurology Foundation, IRCCS, Pavia, Italy, Department of Brain and Behavioural Sciences, University of Pavia, Pavia, Italy; **Eva Azicnuda**, IRCCS Santa Lucia Foundation, Rome, Italy; **Francesco Barale**, Department of Brain and Behavioural Sciences, University of Pavia, Pavia, Italy; **Michelangelo Bartolo**, Neurorehabilitation Unit, IRCCS INM Neuromed, Pozzilli, Italy; **Roberto Bergamaschi**, C. Mondino National Institute of Neurology Foundation, IRCCS, Pavia, Italy; **Mariangela Berlangieri**, Department of Brain and Behavioural Sciences, University of Pavia, Pavia, Italy; **Vanna Berlincioni**, Department of Brain and Behavioural Sciences, University of Pavia, Pavia, Italy; **Laura Berliocchi**, Department of Health Sciences, University Magna Graecia of Catanzaro, Catanzaro, Italy; **Eliana Berra**, C. Mondino National Institute of Neurology Foundation, IRCCS, Pavia, Italy; **Giulia Berto**, Department of Neurological and Movement Sciences, University of Verona, Verona, Italy; **Silvia Bonadiman**, Department of Neurological and Movement Sciences, University of Verona, Verona, Italy; **Sara Bonazza**, Department of Surgery, University of Verona, Verona, Italy; **Federica Bressi**, Campus Biomedico University, Rome, Italy; **Annalisa Brugnera**, Department of Neurological and Movement Sciences, University of Verona, Verona, Italy; **Stefano Brunelli**, IRCCS Santa Lucia Foundation, Rome, Italy; **Maria Gabriella Buzzi**, IRCCS Santa Lucia Foundation, Rome, Italy; **Carlo Cacciatori**, Department of Neurological and Movement Sciences, University of Verona, Verona, Italy; **Andrea Calvo**, Rita Levi Montalcini Department of Neuroscience, University of Turin, Turin, Italy; **Cristina Cantarella**, Physical and Rehabilitation Medicine Unit, Tor Vergata University, Rome, Italy; **Augusto Caraceni**, Palliative Care, Pain Therapy and Rehabilitation, Fondazione IRCCS Istituto Nazionale dei Tumori di Milano, Milan, Italy; **Roberto Carone**, Neuro- Urology Department, City Hospital Health and Science of the City of Turin, Turin, Italy; **Elena Carraro**, Neuropediatric Rehabilitation Unit, E. Medea Scientific Institute, Conegliano, Italy; **Roberto Casale**, Department of Clinical Neurophysiology and Pain Rehabilitation Unit, Foundation Salvatore Maugeri IRCCS, Montescano, Italy; **Paola Castellazzi**, Department of Neurological and Movement Sciences, University of Verona, Verona, Italy; **Gianluca Castelnuovo**, Psychology Research Laboratory, Istituto Auxologico Italiano IRCCS, Ospedale San Giuseppe, Verbania, Italy, Department of Psychology, Catholic University of Milan, Italy; **Adele Castino**, ASL of the Province of Lodi, Lodi, Italy; **Rosanna Cerbo**, Hub Terapia del Dolore Regione Lazio, Policlinico Umberto I, Sapienza University, Rome Italy; **Adriano Chiò**, Rita Levi Montalcini Department of Neuroscience, University of Turin, Turin, Italy; **Cristina Ciotti**, Physical and Rehabilitation Medicine Unit, Tor Vergata University, Rome, Italy; **Carlo Cisari**, Department of Health Sciences, Università del Piemonte Orientale, Novara, Italy; **Daniele Coraci**, Department of Orthopaedic Science, Sapienza University, Rome, Italy; **Elena Dalla Toffola**, Department of Clinical, Surgical, Diagnostic and Pediatric Sciences, University of Pavia, Pavia, Italy, IRCCS Policlinico San Matteo Foundation, Pavia; **Giovanni Defazio**, Department of Basic Medical Sciences, Neuroscience and Sensory Organs, Aldo Moro University of Bari, Bari, Italy; **Roberto De Icco**, C. Mondino National Institute of Neurology Foundation, IRCCS, Pavia, Italy, Department of Brain and Behavioural Sciences, University of Pavia, Pavia, Italy; **Ubaldo Del Carro**, Section of Clinical Neurophysiology and Neurorehabilitation, San Raffaele Hospital, Milan, Italy; **Andrea Dell'Isola**, Department of Health Sciences, Università del Piemonte Orientale, Novara, Italy; **Antonio De Tanti**, Cardinal Ferrari Rehabilitation Center, Santo Stefano Rehabilitation Institute, Fontanellato, Italy; **Mariagrazia D'Ippolito**, IRCCS Santa Lucia Foundation, Rome, Italy; **Elisa Fazzi**, Childhood and Adolescence Neurology and Psychiatry Unit, City Hospital, Brescia, Italy, Department of Clinical and Experimental Sciences, University of Brescia, Brescia, Italy; **Adriano Ferrari**, Children Rehabilitation Unit, IRCCS Arcispedale S.Maria Nuova, Reggio Emilia, Italy; **Sergio Ferrari**, Department of Neurological and Movement Sciences, University of Verona, Verona, Italy; **Francesco Ferraro**, Section of Neuromotor Rehabilitation, Department of Neuroscience, Azienda Ospedaliera Carlo Poma, Mantova, Italy; **Fabio Formaglio**, Palliative Care, Pain Therapy and Rehabilitation, Fondazione IRCCS Istituto Nazionale dei Tumori di Milano, Milan, Italy; **Rita Formisano**, IRCCS Santa Lucia Foundation, Rome, Italy; **Simone Franzoni**, Poliambulanza Foundation Istituto Ospedaliero, Geriatric Research Group, Brescia, Italy; **Francesca Gajofatto**, Department of Neurological and Movement Sciences, University of Verona, Verona, Italy; **Marialuisa Gandolfi**, Department of Neurological and Movement Sciences, University of Verona, Verona, Italy; **Barbara Gardella**, IRCCS Policlinico San Matteo Foundation, Pavia; **Pierangelo Geppetti**, Department of Health Sciences, Section of Clinical Pharmacology and Oncology, University of Florence, Florence, Italy; **Alessandro Giammò**, Neuro-Urology Department, City Hospital Health and Science of the City of Turin, Turin, Italy; **Raffaele Gimigliano**, Department of Physical and Mental Health, Second University of Naples, Naples, Italy; **Emanuele Maria Giusti**, Department of Psychology, Catholic University of Milan, Italy; **Elena Greco**, Department of Neurological and Movement Sciences, University of Verona, Verona, Italy; **Valentina Ieraci**, Department of Oncology and Neuroscience, University of Turin, City Hospital Health and Science of the City of Turin, Turin, Turin, Italy; **Marco Invernizzi**, Department of Health Sciences, Università del Piemonte Orientale, Novara, Italy; **Marco Jacopetti**, University of Parma, Parma, Italy; **Marco Lacerenza**, Casa di Cura San Pio X S.r.l., HUMANITAS, Milan, Italy; **Silvia La Cesa**, Department of Neurology and Psychiatry, University Sapienza, Rome, Italy; **Davide Lobba**, Department of Neurological and Movement Sciences, University of Verona, Verona, Italy; **Gian Mauro Manzoni**, Psychology Research Laboratory, Istituto Auxologico Italiano IRCCS, Ospedale San Giuseppe, Verbania, Italy, Department of Psychology, Catholic University of Milan, Italy; **Francesca Magrinelli**, Department of Neurological and Movement Sciences, University of Verona, Verona, Italy; **Silvia Mandrini**, Department of Clinical, Surgical, Diagnostic and Pediatric Sciences, University of Pavia, Pavia, Italy; **Umberto Manera**, Rita Levi Montalcini Department of Neuroscience, University of Turin, Turin, Italy; **Paolo Marchettini**, Pain Medicine Center, Hospital San Raffaele, Milan, Italy; **Enrico Marchioni**, C. Mondino National Institute of Neurology Foundation, IRCCS, Pavia, Italy; **Sara Mariotto**, Department of Neurological and Movement Sciences, University of Verona, Verona, Italy; **Andrea Martinuzzi**, Neuropediatric Rehabilitation Unit, E. Medea Scientific Institute, Conegliano, Italy; **Marella Masciullo**, IRCCS Santa Lucia Foundation, Rome, Italy; **Susanna Mezzarobba**, Department of Medicine, Surgery and Health Sciences, University of Trieste, Trieste, Italy; **Danilo Miotti**, Palliative Care and Pain Therapy Unit, Fondazione Salvatore Maugeri IRCCS, Scientific Institute of Pavia, Pavia, Italy; **Angela Modenese**, Department of Neurological and Movement Sciences, University of Verona, Verona, Italy; **Marco Molinari**, IRCCS Santa Lucia Foundation, Rome, Italy; **Salvatore Monaco**, Department of Neurological and Movement Sciences, University of Verona, Verona, Italy; **Giovanni Morone**, IRCCS Santa Lucia Foundation, Rome, Italy; **Rossella Nappi**, Department of Clinical, Surgical, Diagnostic and Pediatric Sciences, University of Pavia, Pavia, Italy, IRCCS Policlinico San Matteo Foundation, Pavia; **Stefano Negrini**, Don Gnocchi Foundation, Milan, Italy, Department of Clinical and Experimental Sciences, University of Brescia, Brescia, Italy; **Andrea Pace**, Neuro-Oncology Unit, Regina Elena National Cancer Institute of Rome, Rome, Italy; **Luca Padua**, Don Gnocchi Foundation, Milan, Italy, Institute of Neurology, Catholic University, Rome, Italy; **Emanuela Pagliano**, Developmental Neurology Unit, C. Besta Neurological Institute Foundation, Milan, Italy; **Valerio Palmerini**, Hub Terapia del Dolore Regione Lazio, Policlinico Umberto I, Sapienza University, Rome Italy; **Stefano Paolucci**, IRCCS Santa Lucia Foundation, Rome, Italy; **Costanza Pazzaglia**, Don Gnocchi Foundation, Milan, Italy; **Cristiano Pecchioli**, Don Gnocchi Foundation, Milan, Italy; **Alessandro Picelli**, Department of Neurological and Movement Sciences, University of Verona, Verona, Italy; **Carlo Adolfo Porro**, Department of Biomedical, Metabolic and Neural Sciences, University of Modena and Reggio Emilia, Modena, Italy; **Daniele Porru**, IRCCS Policlinico San Matteo Foundation, Pavia; **Marcello Romano**, Neurology Unit, Azienda Ospedaliera Ospedali Riuniti Villa Sofia Cervello, Palermo, Italy; **Laura Roncari**, Department of Neurological and Movement Sciences, University of Verona, Verona, Italy; **Riccardo Rosa**, Hub Terapia del Dolore Regione Lazio, Policlinico Umberto I, Sapienza University, Rome Italy; **Marsilio Saccavini**, ASL 2 Bassa Friulana-Isontina, Italy; **Paola Sacerdote**, Department of Pharmacological and Biomolecular Sciences, University of Milano, Milano, Italy; **Giorgio Sandrini**, C. Mondino National Institute of Neurology Foundation, IRCCS, Pavia, Italy, Department of Brain and Behavioural Sciences, University of Pavia, Pavia, Italy; **Donatella Saviola**, Cardinal Ferrari Rehabilitation Center, Santo Stefano Rehabilitation Institute, Fontanellato, Italy; **Angelo Schenone**, Department of Neuroscience, Rehabilitation, Ophthalmology, Genetics, Maternal and Child Health (DiNOGMI), University of Genoa, Genoa, Italy; **Vittorio Schweiger**, Department of Surgery, University of Verona, Verona, Italy; **Giorgio Scivoletto**, IRCCS Santa Lucia Foundation, Rome, Italy; **Nicola Smania**, Department of Neurological and Movement Sciences, University of Verona, Verona, Italy; **Claudio Solaro**, Neurology Unit, ASL3, Genoa, Italy; **Vincenza Spallone**, Department of Systems Medicine, University Tor Vergata, Rome, Italy; **Isabella Springhetti**, Functional Recovery and Rehabilitation Unit, IRCCS Fondazione S. Maugeri, Pavia, Italy; **Stefano Tamburin**, Department of Neurological and Movement Sciences, University of Verona, Verona, Italy; **Cristina Tassorelli**, C. Mondino National Institute of Neurology Foundation, IRCCS, Pavia, Italy, Department of Brain and Behavioural Sciences, University of Pavia, Pavia, Italy; **Michele Tinazzi**, Department of Neurological and Movement Sciences, University of Verona, Verona, Italy; **Rossella Togni**, Department of Clinical, Surgical, Diagnostic and Pediatric Sciences, University of Pavia, Pavia, Italy; **Monica Torre**, IRCCS Santa Lucia Foundation, Rome, Italy; **Riccardo Torta**, Department of Oncology and Neuroscience, University of Turin, City Hospital Health and Science of the City of Turin, Turin, Turin, Italy; **Marco Traballesi**, IRCCS Santa Lucia Foundation, Rome, Italy; **Marco Tramontano**, IRCCS Santa Lucia Foundation, Rome, Italy; **Andrea Truini**, Department of Neurology and Psychiatry, University Sapienza, Rome, Italy; **Valeria Tugnoli**, Neurological Unit, University Hospital of Ferrara, Ferrara, Italy; **Andrea Turolla**, Neurorehabilitation Department, Foundation IRCCS San Camillo Hospital, Venice, Italy; **Gabriella Vallies**, Department of Neurological and Movement Sciences, University of Verona, Verona, Italy; **Elisabetta Verzini**, Department of Neurological and Movement Sciences, University of Verona, Verona, Italy; **Mario Vottero**, Neuro-Urology Department, City Hospital Health and Science of the City of Turin, Turin, Italy; **Paolo Zerbinati**, Neuro- orthopaedic Program, Hand Surgery Department, Santa Maria Hospital MultiMedica, Castellanza, Italy.

## Author's Note

This paper has been written and shared in the Consensus Conference modality.

## Author Contributions

All authors listed have made a substantial, direct and intellectual contribution to the work, and approved it for publication.

## Conflict of Interest Statement

The authors declare that the research was conducted in the absence of any commercial or financial relationships that could be construed as a potential conflict of interest.
